# Supplementary material for: Preoperative Cognitive Impairment and Postoperative Delirium Predict Decline in Activities of Daily Living after Cardiac Surgery—A Prospective, Observational Cohort Study
Source: Geriatrics (Basel). 2020 Oct 3;5(4):69. doi: 10.3390/geriatrics5040069 (PMC7709655; doi:10.3390/geriatrics5040069)
Supplement: Supplementary file 1 [file geriatrics-05-00069-s001.pdf]

Table 1. Comparison of patients' characteristics – Follow-up vs. No Follow-up.

	Had follow-up (*n* = 125)		No follow-up (*n* = 81)		
	Median	IQR	Median	IQR	P
Age (years)	72	(62–77)	71	(64–77)	0.894
Body mass index	27	(25–30)	27	(24–30)	0.895
ADL (points)	42	(37–46)	44	(36–48)	0324
GDS (points)	1	(1–3)	2	(1–3)	0.419
Length of education (years)	11	[10–14]	11	[9–12]	0.035
Total protein (g/Ll)	45*	[41–49]	46*	[42–45]	0.216
Charlson score	2	(1–3)	2	(1–3)	0.314
EuroScore (points)	6	(4–7)	6	(4–8)	0.872
Hemoglobin (g/dL)	13.8	(12.5–14.6)	13.2	(12.2–14.2)	0.061
Platelet count (G/L)	227.0	(183–279)	245.0	(213–277)	0.079
WBC (G/L)	7.2	(5.9–8.5)	6.9	(5.9–8.2)	0.347
CRP (mmol/L)	2.6	(1.3–6.7)	2.8	(1.1–7.1)	0.778
Creatinine (mg/dL)	1.1	(0.9–1.3)	1.1	(0.9–1.3)	0.088
	N	%	N	%	P
Male/female	94/31	75/25%	44/37	54/46%	0.002
MMSE ≤ 26	40	32%	25	31%	0.879
Visual impairment	123	98%	74	91%	0.030
Hearing impairment	27	21%	18	22%	1.000
Atherosclerosis	13	10%	14	17%	0.204
Other than isolated CABG	68	54%	47	58%	0.667
AscE	5	4%	3	4%	1.000
Myocardial infarct	33	26%	20	25%	0.871
LV-EF < 50%	99	79%	52	64%	0.024
NYHA IV	2	2%	3	4%	0.384
Chronic atrial fibrillation	19	15%	14	17%	0.701
Chron. pulmonary disease‡	15	12%	10	12%	1.000
Nicotine					
Never	69	55%	38	47%	
Stopped >2 months	45	36%	32	40%	0.394‡
Ongoing	11	9%	11	14%	
Alcohol					
Never	36	29%	17	21%	
Occasionally	62	50%	13	60%	0.381‡
Moderate	21	17%	10	12%	
Heavy	6	5%	5	6%	
Chron. renal insufficiency‡	14	11%	14	17%	0.220
Diabetes mellitus 2	26	21%	17	21%	1.000
History of malignancy‡	15	12%	5	6%	0.229
History of stroke‡	2	2%	1	1%	1.000
APOEε4 carrier‡	28	25%	22	30%	0.612
	Median	IQR	Median	IQR	P
Duration cardiac bypass (min)	123	[104-149]	117	[99–146]	0.251
Aortic clamp time (min)	77	[60–109]	72	[61–97]	0.316
Duration ≤65mmHg (min)	120	[75–160]	115	[80–160]	0.952
Deepest temperature	34.0	[33.8–34.4]	34.1	[33.9–34.5]	0.049

	N	%	N	%	P
Vasopressors >0.05 μg·kg^{-1}·h^{-1}	101	87%	70	95%	0.136
Milrinone (use of)	16	14%	10	14%	1.000
	Median	IQR	Median	IQR	*p*
SAPS II on admission to ICU	31	(27–36)	32	(26–37)	0.878
Packed red cells	4	[2–6]	3	[2–6]	0.564
Ventilator days	1	(1-2)	1	(1–2)	0.198
Length of stay in ICU	2	(1–4)	2	(1–4)	0.287
Length of stay in hospital	14	(10–21)	13	(9-18)	0.068
ADL/180 days	40	(35–45)	n/a		n/a
MMSE/180 days, telephone	20	(17–22)	19	(16–21)	0.72
	n	%	*n*	%	*p*
Delirium, length of					
0 days	86	69%	57	70%	0.623‡
1 day	16	13%	7	9%	
≥2 days	23	18%	17	21%	
Delirium, subtype					
hypoactive	21	54%	13	54%	0.870
hyperactive	10	26%	5	21%	
mixed	8	20%	6	25%	

Preoperative patients' demographics and risk factors divided into whether they 'Had follow-up or 'No follow-up'.Continuous data were compared with Mann-Whitney U test and presented as median with interquartile range [IQR].Dichotomous data are presented as number and percentage of patients and were compared with two-sided Fisher's exact test or Pearson Chi-Square test, the latter indicated by ‡. ADL, activity of daily living; CRP, C-reactive protein; GDS, geriatric depression scale; MMSE, mini-mental state examination; WBC, white blood cell count; LV-EF, left ventricle ejection fraction; NYHA, classification of heart failure according to the New York Heart Association; APOEε4, carrier of ε4 allele of apolipoproteine E gene; SAPS, Simplified Acute Physiology Score.* Total protein, preoperatively, $n = 60$, postoperatively, $n = 42$ # Preoperative ADL $n = 73$, postoperative ADL $n = 71$.
